# Advocating for trust in and trustworthy AI to transform evidence synthesis

**DOI:** 10.1186/s13750-025-00369-2

**Published:** 2025-10-22

**Authors:** Isabel K. Fletcher

**Affiliations:** Wellcome Trust, London, UK

**Keywords:** Artificial intelligence, Evidence synthesis, Policy, Equity, Data, Technology, Tools

## Abstract

The global demand for high-quality, robust and up-to-date evidence to guide decision-making has never been higher. The vast quantity of scientific literature being produced and made accessible presents an unparalleled opportunity for evidence-based decision-making to become a widespread reality. In addition, the world has at its fingertips cutting-edge technologies, such as AI, to make sense of this extensive knowledge base and deliver insights more quickly to decision-makers most in need. AI-powered evidence syntheses promises to be transformative, saving many lives and enhancing livelihoods globally. However, achieving this requires substantial cultural shifts in the evidence community, including amongst both AI developers and users to shape both trustworthy AI and trust in AI. Current efforts to establish best practices are emerging, but progress is hindered by the lack of clear consensus on what constitutes trustworthy AI for evidence synthesis. Philanthropic investments in trustworthy AI systems, alongside robust evaluations of trust in AI for evidence synthesis, must be prioritised to determine the conditions required for an enabling environment. Mainstreaming AI for reliable, faster and cheaper evidence synthesis demands a better understanding of trustworthy AI and trust in these systems. Funders should prioritise aspects of trustworthiness and trust whilst balancing the drive towards ongoing innovation.

## Introduction

The global evidence ecosystem stands at a pivotal juncture. Compounding global crises, such as climate change, pandemic threats, geopolitical instability and conflict are placing pressure on governments and decision-makers to be responsive in light of robust evidence. At the same time, the rapid rise of artificial intelligence (AI) capabilities and its integration into daily life [2] and sectors including healthcare [26], education [64] and the environment [25] promise significant transformations in evidence-based decision making, particularly for evidence synthesis. However, the provisioning of robust policy-relevant evidence assisted by AI can only be achieved using both trustworthy AI systems and by unlocking vital components of user trust.

Recent calls to action to transform the current state of global policymaking, such as that through the publication of the SHOW-ME framework [34], have signalled a readiness of the global evidence community to reform how evidence, including syntheses, are developed and delivered to address the most pressing global needs, such as the stalling progress of the Global Sustainable Development Goals. Philanthropic efforts have acknowledged these calls to action, by addressing the need for more coordinated evidence synthesis infrastructure and capacity sharing [27], [63], alongside enhancing the policy-relevance of AI applications [44, 57]. While strong signals from the community, complemented by funder support, present valuable opportunities to advocate for evidence-based decision making, the contested role of AI and emerging technologies is hindering transformational progress and a multitude of opportunities. This is in part due to an absence of consensus within the community on the characteristics of trustworthy AI for evidence synthesis and the practical implementation required for widespread adoption, which facilitates trust. The global evidence community must now decide how to effectively meet the demand for evidence-based policy and foster a cultural shift towards trustworthy AI, whilst simultaneously balancing the opportunities and risks of AI, which are rapidly reshaping the generation, synthesis and use of evidence.

### A dynamic landscape of AI opportunities and challenges

Evidence syntheses are a critical policy tool, which consolidate knowledge across a range of sources using a transparent methodology, and include systematic reviews, meta-analyses and scoping reviews [53]. Synthesis of evidence is crucial for making diverse forms of knowledge useful, navigating large bodies of evidence and enabling policy-relevant action that is relevant across multiple local and national scales.

There are vast technological opportunities for AI to assist in the production and delivery of evidence syntheses for impactful decision-making, many of which have already been demonstrated, applied and widely used in various tooling solutions [29]. AI is the capability of machines or systems to do tasks that usually require human intelligence or input [51]. Applications of AI for evidence synthesis have primarily centered around automation of elements in the production of a systematic review, such as literature searching, abstract screening and data extraction. For example, active machine learning techniques have been employed to improve screening efficiency, whilst not compromising on quality [58]. Large language models (LLMs) are a class of AI capable of generating natural language text in response to user inputs [49, 60], and for evidence synthesis can be customised to retrieve and evaluate policy options [6, 50]. In many cases AI has been used as a supplement to manual labour efforts, such as to provide living updates of evidence through continuous study identification [37] and to prioritise screening in systematic reviews [11], with higher recall and lower effort than conventional methods [48]. In addition, machine learning techniques have been employed successfully to track dynamic, rapidly evolving bodies of evidence, such as that on global climate policies [50] and COVID-19 [47]. These methods have been used effectively to address the challenge of ‘big literature’ [40] and can help define synthesis topics while also highlighting knowledge gaps.

The latter stages of the synthesis and review process are less well exploited by AI [12], [62]. This includes data extraction for systematic reviews, which remains a labour-intensive task, indicating that further work is needed to train AI models to incorporate contextual and culturally-sensitive interpretations from texts [6]. Amongst the landscape of rapid emerging AI-tools for evidence synthesis it remains difficult for users, particularly those with less experience to select the best AI-assisted solution [62]. This is in part due to the absence of robust evaluations of these tools [24], [62], which contributes to the slow adoption of AI for evidence synthesis, creating an adoption divide where only AI-experienced users benefit from the resulting efficiency gains.

Integration of AI into evidence synthesis workflows has enhanced the feasibility of ‘living systematic reviews’ (LSR), whereby reviews are continually updated and incorporate relevant new evidence as it becomes available [16, 17, 55]. LSRs have proliferated and have become widely accepted methodology. This has been particularly useful in areas where policy-demand is high and the evidence base is highly dynamic [65]. LSRs also present an effective model for upholding the rigour of scientific evidence and are particularly impactful in fields where it is crucial to keep pace with rapidly emerging bodies of evidence, such as during the COVID-19 pandemic [18].

However, LSRs have yet to be integrated into the mainstream culture of evidence synthesis production and delivery. LSRs pose significant barriers to entry and use, requiring specialist training, substantial setup time, and are resource-intensive and complex to manage [9, 39]. Consequently, LSRs may only be adopted where resources and capacity are concentrated. LSR also face dissemination challenges, such as publication delays due to a lack of established and standardised processes to sustain and report LSRs [65]. Trust issues can also arise from inconsistent application of updates and lack of transparency in the inclusion criteria for incorporating new evidence into a LSR. Decision makers may struggle to regard a LSR as rigorous if it isn’t updated, even when new evidence does not emerge that is significant enough to affect policy outcomes.

### Building trustworthy AI

To achieve effective implementation of AI for evidence synthesis, it is important to disentangle what it means to develop a trustworthy AI system, versus how to build trust in an AI system. Trust in AI and trustworthy AI are co-dependent on each other, yet having one does not necessarily guarantee the other. Trustworthy AI is typically thought of as having a transparent, open-access system [8] and is an intrinsic property of the AI system whilst trust is a perception granted by users that is vastly multidimensional [15].

The risks of AI have been extensively documented and disseminated [21] and despite this challenges in developing trustworthy AI continue to inhibit widespread uptake and adoption [2, 22, 41, 45]. In response to global concerns over AI risks, there have been numerous calls for improved safeguards, standards and regulations that balance AI innovation with appropriate risk mitigation. For example, the European Union (EU) has introduced the EU AI Act, which outlines risks of AI and obligations of developers to mitigate such risks [19].

Multiple efforts have attempted to define trustworthy AI, aiming to apply these insights in practice. This has primarily been explored in the medical, healthcare and education sectors, resulting in several tools and guidance to encourage responsible AI practices. In healthcare, various tools and frameworks are used to guide responsible AI practice and reporting, covering areas such as data access, transparency, compliance and accountability [32, 35]. The MedinAI guidelines offer core principles for reporting on AI use in medicine [8], while the DECIDE-AI guidelines assist in evaluating and reporting AI-driven clinical decision support systems to assess performance [59]. In response to the slow adoption of AI in healthcare, the FUTURE-AI framework was developed with interdisciplinary experts, producing six guiding principles to operationalise trustworthy AI: fairness, universality, traceability, usability, robustness, and explainability [36]. However, many of these current guidance frameworks have faced criticism for being overly principle-based, making them difficult for developers to incorporate. To address this, action-oriented approaches, such as the ELATE guidance [14] are beginning to emerge, promising to close the principles-practice gap. In the ELATE guidance for example, a set of guiding questions are proposed for use by developers.

While there is widespread agreement across sectors that AI should be employed with a strong ethical lens, there are competing and inconsistent views on how this should be realised in practice [5, 7, 13, 28]. For example, existing guidelines for reporting AI use in medical research have been criticised for lacking universality and validation in practice [32]. In some cases, where trustworthy AI is applied in fields like healthcare, significant shortcomings remain. Notably, the perspectives of diverse stakeholders [13, 30], close collaboration with AI system developers and end-users [20, 52] are frequently omitted. These findings highlight the significant need to integrate trustworthy AI guidance into existing or new implementation strategies to ensure practical applicability. Recently, the development of the RAISE guidelines [54, 56] has begun to address the unmet need for specific guidance and best practices in applying AI to evidence synthesis.

### Building trust in AI

A key component of building user trust is engineering a transparent system. Rapid technological advancements within a highly competitive landscape have resulted in the untransparent development of many AI systems, with users grappling to fully comprehend so called ‘black boxes’. This has resulted in a limited explainability of AI, further challenging users on the extent to which they should trust AI [3, 52]. For example, it is well documented that AI systems perpetuate existing biases [42, 61] and LLMs can ‘hallucinate’ by providing incorrect or misleading information [4]. The lack of transparency with which many cutting-edge AI systems operate is a significant barrier to trustworthy implementation [49]. In the world of evidence synthesis, this lack of trust in AI manifests as scepticism towards automation replacing humans in labour-intensive tasks, such as data extraction [41], [62].

The evidence synthesis community can gain valuable insights from sectors such as healthcare and education on fostering user trust in AI systems. A significant barrier to AI adoption in evidence synthesis is the reluctance to entrust AI with automating complex tasks such as data extraction [41], [62]. Focusing on which tasks AI should and should not undertake can help address these concerns [23], and can be further mitigated by integrating meaningful human interaction with AI systems through human-in-the-loop procedures [38], such as combining manual screening with machine-learning prioritisation [10, 31]. Another key learning has been the prominent need to acknowledge and adapt to the nuances of trust in AI, as well as the social influences that shape AI use and barriers to adoption. Trust in AI is highly dependent on personal beliefs and user experience, alongside both perceived and actual developer expertise [1, 7, 15, 33]. Therefore, the assumption that transparent and open AI systems are trusted by users is problematic. While transparency has been shown to be a major facilitator of trust in AI, it may in some instances lessen perceived user risks of AI, thereby reducing the need for trust in AI [46]. These complex issues around trust therefore have practical implications for how AI systems are used responsibly and highlight the need for ongoing meaningful user engagement [15].

### The role of funders

A reform of the global evidence ecosystem to develop and use trustworthy AI-powered evidence synthesis requires a careful balance of innovation alongside a cultural shift to embed best practices. The development of trustworthy AI and fostering trust in resulting systems must be prioritised in philanthropic discourse, allowing for new communities of practice to emerge. Given the growing concern over the risks of AI, it is no longer sufficient to continue to focus solely on development innovation to achieve quick technological wins. Funders must prioritise the development of trustworthy AI systems to support and accelerate synthesis of all and diverse forms of evidence, alongside robust evaluation in practice to ensure AI-assisted synthesis workflows are fit for purpose. Addressing trustworthy AI and trust in AI separately will allow funders to more effectively assess the impact of their investments and target programmes to prop up trust where it is lacking. Furthermore, funders should set expectations to develop AI tools and systems in an open access environment by providing incentives and direct funding to do so, integrating transdisciplinary methods of participatory design and evaluation, and fostering direct accountability and ownership of AI tools to developers to increase transparency. To address the contextual complexities of evidence use and increase uptake of synthesis products, funders should invest in purpose-built AI-assisted policy instruments or adapt existing AI tools for evidence synthesis to target evidence demand in particular policy fields. For example, AI-generated summaries can be an efficient resource to support evidence-based policymaking, ensuring evidence is both useful and used.

It is evident that guidelines for the development and use of AI for evidence synthesis are crucial, and while progress is being made, greater support is required to enhance their uptake and long-term sustainability. Funders should support collaborative impact mechanisms that enable multiple sectors, disciplines and fields of expertise to co-produce guidance and frameworks that can be used across the evidence synthesis ecosystem to address unmet demand. Importantly, this should be done in a way so as not to stifle innovation or discourage AI use due to the challenges in overcoming risks and concerns over trust in AI. Direct support for the co-development and application of practical guidance (e.g. [38, 54, 56]) along with iterative testing and evaluation with multiple stakeholders should be prioritised. In addition, what is now needed is a thorough evaluation and critical appraisal of such guidelines in practice to further refine their usability. All guidance developed should be underpinned by empirical evidence, which champions widespread community acceptance and adoption.

Currently, some policymakers are not making the best use of evidence [43] and there is great potential for AI to deliver faster, cheaper evidence syntheses in areas where evidence is unavailable and underutilised. AI will be transformational for policymaking, but only if the need for improved integration with policymaking and evidence demand is met [54, 56]. This would also enable demands for evidence to be adapted under rapidly evolving conditions, such as that witnessed during the COVID-19 pandemic. Exploring opportunities for AI to more directly aid the evidence-to-decision workflow is another gap funders could address in the short-term to enhance the useability of AI-assisted synthesis. The potential for AI-powered evidence synthesis to be transformative is unparallelled and the immediate challenge is to enable this transformation in a trustworthy way that secures a sustainable cultural shift towards best practices.

## Data Availability

No datasets were generated or analysed during the current study.
